# The Potential of Carnosine in Brain-Related Disorders: A Comprehensive Review of Current Evidence

**DOI:** 10.3390/nu11061196

**Published:** 2019-05-28

**Authors:** Martin Schön, Aya Mousa, Michael Berk, Wern L. Chia, Jozef Ukropec, Arshad Majid, Barbara Ukropcová, Barbora de Courten

**Affiliations:** 1 Institute of Pathophysiology, Faculty of Medicine, Comenius University, 84215 Bratislava, Slovakia; martin.schon01@gmail.com; 2 Biomedical Research Center, Slovak Academy of Sciences, 81439 Bratislava, Slovakia; jozef.ukropec@savba.sk; 3 Monash Centre for Health Research and Implementation, School of Public Health and Preventive Medicine, Melbourne, VIC 3168, Australia; aya.mousa@monash.edu (A.M.); wernchia87@gmail.com (W.L.C.); 4 School of Medicine, IMPACT Strategic Research Centre, Barwon Health, Deakin University, Geelong, VIC 3220, Australia; michael.berk@barwonhealth.org.au; 5 Orygen, The Centre of Excellence in Youth Mental Health, the Department of Psychiatry and the Florey Institute of Neuroscience and Mental Health, The University of Melbourne, Melbourne, VIC 3052, Australia; 6 Sheffield Institute for Translational Neuroscience, University of Sheffield, Sheffield S10 2HQ, UK; arshad.majid@sheffield.ac.uk; 7 Faculty of Physical Education and Sports, Comenius University, 81469 Bratislava, Slovakia

**Keywords:** carnosine, L-histidine, β-alanine, brain, cognition, treatment, psychiatry, neurology, nervous system

## Abstract

Neurological, neurodegenerative, and psychiatric disorders represent a serious burden because of their increasing prevalence, risk of disability, and the lack of effective causal/disease-modifying treatments. There is a growing body of evidence indicating potentially favourable effects of carnosine, which is an over-the-counter food supplement, in peripheral tissues. Although most studies to date have focused on the role of carnosine in metabolic and cardiovascular disorders, the physiological presence of this di-peptide and its analogues in the brain together with their ability to cross the blood-brain barrier as well as evidence from in vitro, animal, and human studies suggest carnosine as a promising therapeutic target in brain disorders. In this review, we aim to provide a comprehensive overview of the role of carnosine in neurological, neurodevelopmental, neurodegenerative, and psychiatric disorders, summarizing current evidence from cell, animal, and human cross-sectional, longitudinal studies, and randomized controlled trials.

## 1. Introduction

Brain disorders represent a serious threat to human health because of both their high prevalence, which continues to rise in line with increasing life expectancy, as well as their associated disabilities, heavy economic burden, and lack of effective and tolerable treatments [1]. According to a World Economic Forum report [1], the global percentage of individuals aged more than 60 years will double from 11% in 2010 to 23% in 2050. Consistent with aging of the population, cardiovascular diseases, neurodegenerative conditions, and mental health conditions have now become the dominant contributors to the global burden of non-communicable diseases (NCDs). In fact, mental health conditions are now the leading cause of Disability Adjusted Life Years (DALYs), accounting for 37% of healthy life years lost from NCDs, and their global cost is expected to surge from $2.5 trillion USD in 2010 to $6.0 trillion USD by 2030 [1]. Neurodegenerative and neuropsychiatric conditions are usually treated symptomatically and currently available drugs generally lack disease-modifying activity, have low efficacy, and/or significant tolerability burdens [2,3,4,5,6]. Hence, there is an urgent need to identify more effective, low-cost, and easily scalable interventions to prevent and treat neurological, neurodegenerative, and psychiatric disorders.

A growing body of evidence indicates that exercise is effective in the prevention and treatment of various chronic disorders (reviewed in Reference [7]), including neurodegenerative and neuropsychiatric conditions. A dipeptide, carnosine (β-alanine-L-histidine), was identified as an exercise enhancer and has been widely used in sports with the aim of improving physical performance and muscle gain [8]. Carnosine has been shown to favourably affect energy and calcium metabolism, and reduce lactate accumulation [9,10]. Notwithstanding the biochemical complexity of exercise, both exercise and carnosine may exert similar effects including optimization of energy metabolism, improvement of mitochondrial function, and reduction of systemic inflammation, and oxidative stress [11,12,13]. Although 99% of carnosine in the human body is located in skeletal muscle, carnosine is also present in heart muscle as well as in specific areas of the brain at approximately 100-fold lower concentrations [10,12]. Thus, carnosine is found primarily in the two tissues with the most active oxidative metabolism, which are tissues in muscles and the brain. Both of carnosine’s precursors, β-alanine and L-histidine, can be easily taken up from circulation into the brain through amino acids transporters in the blood-brain barrier (BBB) [14]. This enables local carnosine synthesis in the brain, which takes place in olfactory neurons [15] and in glial cells, specifically in mature oligodendrocytes [16,17]. Carnosine itself can also cross the BBB [18], but it is thought that the majority of brain carnosine is a product of its de novo synthesis localized to specific areas of the brain rather than a result of its penetration through the BBB [12]. Carnosine together with homocarnosine, which is a dipeptide of gamma-aminobutyric acid (GABA) and histidine and the dominant carnosine analogue in the human brain, are both present in cerebrospinal fluid (CSF) [16].

The presence of carnosine and its analogues in the brain suggests that these histidine-related compounds may play some physiological role in brain function, as endogenous antioxidants, neuromodulators, and neuroprotective molecules [12]. However, despite a number of studies demonstrating the anti-ischemic and neuroprotective properties of carnosine, there is currently no unified hypothesis as to the exact role of carnosine in brain disorders, or its potential use in preventing or managing these conditions. Although previous reviews including systematic reviews and meta-analyses on this topic have been conducted, these tend to focus on specific disorders such as neurodegenerative disorders [19] or depression [20], or are limited to human studies, overlooking the large body of evidence derived from experimental and animal models. Given these limitations and the considerable number of newly published studies, a comprehensive updated review of the evidence in relation to carnosine and brain-related disorders is pertinent.

In this narrative literature review, we aimed to summarize current evidence regarding the potential role of carnosine in brain-related disorders, including neurological, neurodevelopmental, neurodegenerative, and psychiatric disorders from cell, animal, and human studies including clinical trials and meta-analyses. We did not intend to introduce new data or conclusions but rather to integrate and contextualise the current state of knowledge in this area and to identify relevant evidence gaps. For the purpose of this review, we define neurological disorders as those conditions with recognisable pathological damage to the brain (e.g., ischemia/stroke), neurodevelopmental disorders as abnormal brain development (e.g., Autistic spectrum disorders), neurodegenerative disorders as involving cell death and degeneration over time (e.g., Alzheimer’s, Parkinson’s), and psychiatric disorders as those which affect mental functioning and behaviour (e.g., schizophrenia, mood disorders). We searched relevant publications in PubMed using the following keywords without date limits including both clinical and preclinical data: carnosine, β-alanine, L-histidine, anserine, dementia, cognition, Alzheimer disease, mild cognitive impairment, Parkinson disease, multiple sclerosis, stroke, brain ischemia, brain hemorrhage, brain trauma, epilepsy, Autistic spectrum disorders, mood disorders, anxiety, depression, schizophrenia, Attention-deficit/hyperactivity disorder, obsessive-compulsive disorder, post-traumatic disorder, and dyslexia.

## 2. Proposed Mechanisms Mediating the Role of Carnosine in the Brain

**Figure 1:**
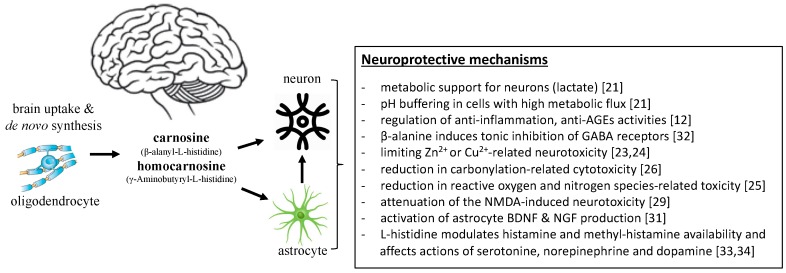
Proposed mechanisms mediating the neuroprotective effects of carnosine in the brain.

Caruso et al. [21] posits that the role of carnosine in brain-related disorders is analogous to its role in skeletal muscle. Neurons and astrocytes are thought to be the main utilisers of brain carnosine [16]. In astrocytes, carnosine was shown to facilitate lactate export from cells and, hence, provide metabolic support for neurons and axons by buffering protons [21] (Figure 1). Furthermore, well-known peripheral actions of carnosine were also reported in the brain, such as reductions in oxidative stress, inflammation, and advanced glycation end products as well as regulation of macrophage function (reviewed in detail in Reference [12]). Carnosine is also a precursor to a key inhibitory neurotransmitter in the brain, GABA [22], and can act as a Zn^2+^ and Cu^2+^ chelator and, thus, exert neuroprotective action on Zn^2+^ and Cu^2+^-mediated neurotoxicity [23,24] (Figure 1).

Carnosine exerts its anti-oxidative actions indirectly through modulation of the endogenous anti-oxidant system as well as directly by decreasing the intracellular levels of reactive species, such as hydroxyl radicals, nitric oxide [25], and cytotoxic carbonyl species [26] (Figure 1). This was confirmed in a study in which administration of carnosine to rat cerebellar cells halved ROS concentrations in neurons [27]. Moreover, carnosine has anti-oxidative capacity against agents increasing ROS [28]. Another neuroprotective action of carnosine includes its attenuation of neurotoxicity induced by NMDA [29], beta amyloid, and inducible nitric oxide synthase [30] as well as increased trophic factors expression [31] (Figure 1). The effects of carnosine were reversed by inhibiting histidine decarboxylase (HDC) as well as histamine H_1_ and H_3_ antagonists, which suggests that carnosine’s neuroprotective mechanisms include not only the carnosine-histidine-histamine pathway, but also H_1_ and H_3_ receptors [29].

When investigating the role of carnosine in brain function and brain-related disorders, one must take into consideration the existing evidence regarding carnosine precursors and derivatives. β-alanine is considered as a neurotransmitter with the ability to reverse the blocking actions of GABA receptors [32]. L-histidine is involved in regulating food intake and neurotransmitters serotonine, dopamine, and norepinephrine [33] (Figure 1). Carnosine derivatives anserine and N-acetyl carnosine are proposed to be more potent than carnosine itself, possibly due to their resistance to enzymatic hydrolysis [28,30]. Lastly, carnosine is recognized as a non-mast cell reservoir for histidine, utilized for the synthesis of histamine [34], which is an important neuromodulator with a role in brain disorders likely mediated by the type of histamine receptors. This probably accounts as an important mechanism of carnosine actions in the brain (Figure 1).

## 3. Cell Studies of the Role of Carnosine in Brain-Related Disorders

### 3.1. Neurological Disorders

#### Brain Ischemia

Several in vitro models of rodent brain cells have been used to examine the potential neuroprotective and anti-ischemic effects of carnosine in the brain. Brain ischemia is a common disorder affecting over 15 million people every year worldwide and occurs when the oxygen supply to the brain is interrupted [35]. Brain ischemia leads to alteration of astrocytes, including their hypertrophy and increased capacity for proliferation, migration, and production of pro-inflammatory cytokines [36]. Pre-treatment of primary cultured astrocytes with carnosine after exposure to oxygen-glucose deprivation suppressed proliferation and migration of reactive astrocytes and enhanced glycolysis and ATP production [37]. This resulted in the reduction of ischemic cell death, attenuation of autophagic signaling, and improvement of mitochondrial function [38,39,40]. These mechanisms might also explain carnosine–related reduction of infarction volume in an in vivo rat model of focal ischemic stroke [40]. Moreover, carnosine treatment rescued the expression of glutamine synthetase (GS) and reversed glutamate uptake activity and production of glutamine in the senescent astrocytes exposed to oxygen-glucose deprivation/recovery (OGD/R) [41]. Furthermore, carnosine treatment dose-dependently decreased the number of apoptotic microvascular endothelial cells in culture [42] and restored the activity of glutaminergic and GABA-ergic (gamma-aminobutyric acid) receptors in cortex slices in a model of hemorrhage stroke, which reduced swelling of brain tissue damaged by blood clots [43].

The effects of carnosine on astrocytes were abolished by histamine H_1_, but not H_2_ or H_3_ receptor antagonists, and pretreatment with neither histidine nor histamine exerted the same neuroprotective effect [41,44]. This indicates that the protective effects of carnosine may be mediated by activity at the astrocyte histamine type 1 receptor. However, the effects of carnosine on brain endothelial cells were diminished by adding histamine H_1_ and H_2_ receptor antagonists as well as a selective histidine decarboxylase inhibitor [42], which suggests that the anti-apoptotic effects of carnosine on brain endothelial cells are mediated at least in part through histamine H_1_ and H_2_ receptors.

### 3.2. Neurodegenerative Disorders

#### 3.2.1. Alzheimer’s Disease

A number of in vitro studies have explored the potential role of carnosine in modulating elements of neurodegenerative disorders, especially the formation of β-amyloid fibrils as a pathological hallmark of Alzheimer’s disease (AD). AD is the most common form of dementia and reflects progressive cognitive impairment sufficient to impact on activities of daily living [45]. Atomic force microscopy revealed that carnosine in a dose-dependent fashion reduced amyloid beta peptide 1-42 (Aβ1-42) polymerization and decreased the number of deposited aggregates in a model of the amyloidogenic peptide fragment Aβ1-42, which suggests that carnosine may inhibit Aβ1-42 fibrillogenesis. Carnosine also significantly affected the structure of fibrils with predominance to short-sized fibrillar aggregates and reduced fibril growth [46]. Another study extended these findings by showing that carnosine inhibited amyloid growth. At 20-fold molar excess, carnosine reduced the aggregation of Aβ42 by 70% due to its interactions with the central hydrophobic area of β-amyloid, which plays an essential role in aggregation processes [47]. However, carnosine failed to modify the conformational features of Aβ42 [47] or the pathology of tau protein, which is a protein that stabilizes microtubules that were found to be defective in AD [48].

Carnosine seems to be effective in protecting rat brain vascular endothelial cells (RBE4) against β-amyloid-induced cytotoxicity [49] and to inhibit lysozyme fibril formation-related cytotoxicity in human neuroblastoma SH-SY5Y cells [50]. Another important observation using this neuroblastoma cell model indicated that carnosine co-treatment (but not pretreatment) prevented serotonin-derived melanoid toxicity, which suggests that direct interaction of carnosine with the process of sevoflurane-induced sequestration of age-related acrolein lead to accumulation of serotonine derived melanoid and subsequent neuronal toxicity could be prevented by L-carnosine [51]. Carnosine also induced expression of the brain-derived neurotrophic factor (BDNF) and nerve growth factor (NGF) in a human primary glioblastoma cell line (but not neuroblastoma) [31]. These neurotrophins and their signaling pathways play a critical role in neurodevelopment and adult brain plasticity, neuronal survival, differentiation and function, and repair mechanisms, and have been implicated in aging processes and the development of AD [52]. Another mechanism may be related to carnosine Cu^2^/Zn^2+^ chelating properties. Zn^2+^ and Cu^2+^ play a role in the pathogenesis of AD, since the interaction between β-amyloid and Cu^2+^ results in reactive oxygen species (ROS) formation and release of pre-synaptic Zn^2+^ from glutaminergic terminals, which, in turn, promotes formation of β-amyloid plaques [53]. Two in vitro studies showed that carnosine has inhibitory effects on β-amyloid aggregation and oxidative stress due to chelation of Cu^2^/Zn^2+^ [48,54].

#### 3.2.2. Parkinson’s Disease

Another common neurodegenerative disorder is Parkinson’s disease (PD), which affects ~1% of the population aged more than 60 years [55]. PD is caused by the loss of dopaminergic neurons, which leads to reduced control of smooth muscle and body movements and manifests as tremor, rigidity, and bradykinesia [55]. Only one study has explored the effects of carnosine on a cell model of PD. In this scenario, carnosine reduced apoptosis and mitochondria-derived production of ROS in brain endothelial cells and normalized the levels of lipid peroxidation (malondialdehyde, MDA) and anti-oxidant enzymes [56].

### 3.3. Psychiatric Disorders

#### Major Depressive Disorder

Few cell studies have explored the role of carnosine in pathophysiological models of major depressive disorder (MDD), which is a severe and debilitating form of depression affecting approximately 5–20% of the worldwide population [57]. Recent meta-analyses showed a negative association between depression and telomere length [58] as well as between depression and the presence of metabolic syndrome [59]. Although limited, data from human and animal cell studies suggest that carnosine might have a potential to maintain telomere length [60] and delay cellular senescence [61] in fibroblasts, as well as ameliorate stress-induced changes in skeletal muscle and liver metabolism [62]. These mechanisms are concordant with some of the beneficial effects of carnosine on depressive disorders [20].

### 3.4. Summary of Evidence from Cell Studies

For neurological disorders such as brain ischemia, evidence indicates that the neuroprotective and anti-ischemic effects of carnosine may be specific to cell type and include attenuation of astrogliosis, restoration of cell energetic balance, mitochondrial protection, and reversal of deleterious autophagic processes with a positive impact on the glutaminergic system. Regarding neurodegenerative disorders, evidence from cell studies using in vitro models of AD and PD highlight the functional as well as structural anti-aggregating effects of carnosine in relation to early stage fibril formation, stimulation of neurogenesis in glial cells, and cyto-protection through ion chelation and anti-oxidation, which is likely to take place during cell differentiation. These effects appear to be concentration-dependent and time-dependent [50]. Lastly, cell studies in psychiatric disorders are scarce. Yet, despite indirect and limited evidence, some data support a role for carnosine in factors associated with MDD such as telomere length and cellular senescence, which are intriguing findings that require further confirmation.

## 4. Animal Studies of the Role of Carnosine in Brain-Related Disorders

### 4.1. Neurological and Neurodevelopmental Disorders

#### 4.1.1. Brain Ischemia

Multiple animal studies have explored the mechanisms of carnosine-induced neuroprotection in brain ischemia. A meta-analysis of eight studies [63] analyzing the effects of carnosine on ischemic stroke revealed an average reduction in infarct volume of 29.4% with a clear dose-dependent effect (38.1% reduction on 1000 mg/kg dose compared with 13.2% for doses less than 500 mg/kg). The reductions in infarct size were comparable between administration of carnosine at 30 min prior to ischemia and within 6 h after ischemia [63]. Moreover, neuroprotective carnosine actions were present in both permanent and transient ischemic animal models and the functional and motor improvements were sustained long-term [40,64].

A number of mechanisms have been proposed to explain the effects of carnosine on brain ischemia. Two-week pre-treatment with a formula NT-020 (a pill-based nutraceutical that contained a proprietary formulation of blueberry, carnosine, green tea, vitamin D3, and Biovin) before transient occlusion of the middle cerebral artery in rats reduced motor asymmetry and neurological dysfunction and decreased the ischemic area in the striatum. This was accompanied with a one-fold and three-fold increase in neurogenesis in the subventricular zone and ischemic striatal penumbra, respectively, compared to placebo supplemented rats. These effects may be attributed to growth factors including BDNF, glial cell-derived neurotrophic factor (GDNF), the stem cell factor (SCF), and the vascular endothelial growth factor (VEGF) [65]. However, some authors of this study were involved in commercializing this formula. Another mechanism relates to the anti-inflammatory properties of carnosine, whereby, in a model of permanent cerebral ischemia, supplementation with a beef decoction (in which carnosine accounts for 63% of amino acid content) dose-dependently down-regulated the expression of proinflammatory cytokines interleukin (IL)-6, the tumor necrosis factor-α (TNF-α) and interferon-γ (IFN-γ), and up-regulated the expression of anti-inflammatory cytokine IL-4 [66]. Carnosine pre-treatment before brain ischemia has also been shown to reduce lipid peroxidation and enhance anti-oxidant activity in the hippocampus and prefrontal cortex [67], inhibit mRNA expression of mediators of apoptosis (apoptosis-inducing factor, AIF, caspase 3) [68], and up-regulate mRNA expression of STAT3, which is an important regulator of anti-apoptotic factors [69]. Furthermore, carnosine analogues, anserine (β-alanyl-3-methyl-L-histidine), and N-acetyl carnosine, as well as the carnosinase inhibitor (bestatin), failed to reduce infarct volume or improve neurologic function in a mouse model of focal cerebral ischemia [70].

Several studies have also examined the involvement of histaminergic circuits in carnosine-induced neuroprotection in ischemic stroke. Acute administration of L-histidine in combination with diphenhydramine (an inhibitor of histamine-inactivating enzyme in the brain) reduced infarct size by 25% and improved ischemia-induced brain edema [71]. Favourable effects of L-histidine administration were observed immediately, after 6 hours, and after 24 h upon acute brain ischemic injury [72]. Administration of the H_2_ antagonist ranitidine completely abolished the anti-ischemic benefits of histidine, in contrast to the administration of mepyramine, which is an H_1_ antagonist [73]. This contradicts findings from another study where the neuroprotective effects were abolished by pretreatment with a histamine H_1_ receptor blocker [74]. In another model of permanent cerebral ischemia, carnosine reduced glutamate levels and preserved the expression of glutamate transporter-1 (GLT-1) in astrocytes, which results in reduced glutamate excitotoxicity [75]. This suggests that the anti-ischemic actions of carnosine may also be mediated by GLT-1.

#### 4.1.2. Epilepsy

Some evidence from animal studies suggests that the brain histaminergic system may be involved in epilepsy, which is a chronic neurological disorder with recurrent paroxysmal changes caused by abnormalities in the electrical activities in the brain, which affects 1% of the worldwide population [76]. The central histaminergic system was shown to play a role in the inhibition of convulsions [77], while a diet poor in L-histidine-containing meals raised the susceptibility for seizures in rodents [78]. Animals supplemented for 45 days with a compound composed of homocarnosine, carnosine, and anserine were found to have substantially increased anti-convulsant activity after induction of epilepsy with pentylenenetrazol (PTZ), as well as reduced lipid peroxidation and increased concentrations of antioxidants [79]. Other studies supported the notion that carnosine could act as an anticonvulsant, with reports that carnosinedose-dependently reduced seizure severity and prolonged the latency for myoclonic jerks after induction of seizures [80,81,82,83]. In addition, carnosine increased histamine levels in the hippocampus and cortex, which were decreased after administration of PTZ [80,81]. The carnosine precursor L-histidine itself was shown to be a beneficial adjuvant to the anti-epileptic drugs carbamazepime and phenytoin in mouse models of maximal electroshock-induced seizures [84]. Apart from the carnosine-histidine-histamine pathway, carnosine can also act directly on CA1 pyramidal neurons [85] or as a precursor for the neurotransmitter GABA [86] and, thus, mediate its anti-convulsant properties.

#### 4.1.3. Autism Spectrum Disorder

Autism spectrum disorder (ASD) refers to a cluster of neurodevelopmental conditions characterized by impaired social communication and interaction, and repetitive patterns of interests, behaviours, and activities [87]. Autistic-like behavioral impairments in mice were associated with enhanced phosphorylation of mTOR downstream proteins in the prefrontal cortex and amygdala [88]. Importantly, histidine-containing diet was shown to reduce the phosphorylation of p70 S6K1, which is the downstream target of mTOR in these brain regions in mice, in parallel with the reduction of autistic-like behavior [89]. One unique study examined the effects of dietary histidine on autistic-like behavioral deficits in mice [89]. In vitro histidine was identified as a key regulator of the mammalian target of rapamycin (mTOR) signaling pathway, which has been implicated in the pathogenesis of ASD as well as psychiatric disorders such as bipolar disorder and depression [90,91]. Based on this, an mTOR-targeting amino acid diet rich in histidine was constructed. The dietary intervention normalized autistic-like behavioural deficits in mice, which was attributed to attenuated mTOR signaling in the prefrontal cortex and amygdala [89]. Carnosine has an inhibitory effect on mTOR signaling reducing the phosphorylation of mTOR, Akt, and p70S6K [92].

#### 4.1.4. Multiple Sclerosis

Multiple sclerosis (MS) is a chronic autoimmune and inflammatory disease of the CNS with progressive demyelinization [93]. In an animal model of multiple sclerosis, muscle carnosine levels were decreased and were not restored by exercise, which possibly mirrors altered muscular functions in MS [94]. Another animal study reported delayed onset of symptoms in exercising rats following the induction of MS [95].

### 4.2. Neurodegenerative Disorders

#### 4.2.1. Alzheimer’s Disease

There is a growing body of evidence showing that supplementation with carnosine, anserine, or its precursors could counteract AD pathogenesis at the level of the formation of neurotoxic proteins. Carnosine supplementation in AD might seem reasonable because mice with AD and mice with knockout of beta secretase 1, which is a key enzyme in the clearance of β-amyloid, have reduced carnosine brain content [96]. Moreover, hippocampal β-alanine levels were shown to be important in the modulation of spatial memory [97]. Studies report that carnosine supplementation reduced β-amyloid accumulation in the hippocampus of a transgenic mouse model of AD [48] and eliminated α-crystallin amyloid fibril formation in a rodent model of senile cataract disease [98]. However, supplementation with anserine had no effect on β-amyloid accumulation or plaque formation [99]. Other studies have shown that carnosine [48,100], anserine [99], and L-histidine [101] effectively ameliorated cognitive deficits in contrast to β-alanine [102], which had no effect.

Metabolic disturbance plays an important role in AD pathogenesis, and this is supported by findings that advanced glycation end-products (AGEs) that were found in β-amyloid plaques [103] and pre-incubation of β-amyloid with carbohydrates promoted transformation of soluble β-amyloid into insoluble deposits, which accelerates β-amyloid aggregation [104]. Several studies posit that the mechanisms of carnosine in neurodegeneration may overlap with its mechanisms in metabolism. These mechanisms include reducing oxidative stress [105] with concomitant strengthening of anti-oxidant system [106] and anti-inflammatory response or enhanced activity of acetylcholinesterase [107]. AD is associated with decreased mitochondrial biogenesis, which is a characteristic shared by many neurological and psychiatric disorders [108]. In streptozotocin-treated rodents (a well-established murine model of type 2 diabetes, T2D), carnosine treatment improved glucose metabolism as well as spatial recognition memory, retention, and recall, and prevented reduction of step-through latency in a dose-dependent manner [109]. Furthermore, administration of high-dose carnosine in mice reduced hippocampal acetylcholinesterase and nuclear factor kappa-B activity (NF-kB, a key pathway for induction of brain oxidative stress and inflammation) [109], reduced levels of glial fibrillary acidic protein (GFAP, a marker of astrogliosis in the hippocampus), lowered lipid peroxidation, rescued AD-related mitochondrial dysfunction in the hippocampus and cortex [48], and increased anti-oxidant activity [110,111]. Carnosine treatment also prevented cognitive decline induced by a high fat diet in a rodent model of AD [112].

#### 4.2.2. Vascular Dementia

Vascular dementia, which is the second most common cause of dementia, refers to a cognitive impairment resulting from a chronic brain ischemia in specific brain areas [113]. In a rodent model of ischemic vascular dementia [114], carnosine treatment ameliorated white matter lesions and improved cognitive impairment in wild-type mice as well as in histidine decarboxylase knockout mice, whereas administration of histidine did not show the same effect. Carnosine also suppressed the activation of microglia and astrocytes and attenuated the increase in ROS. Hence, it seems plausible that, in this model, carnosine exerts neuroprotective effects independently of the histaminergic pathway, but due to a suppression of ROS generation, glia activation, and myelin degeneration [114]. Protective effects on neurovascular units were also demonstrated with anserine administration, which resulted in improved pericyte coverage on endothelial cells in the hippocampus and cerebral cortex and diminished glial neuroinflammation in the brains of rats with AD [99].

#### 4.2.3. Parkinson’s Disease

Administration of neurotoxic substances or knock-out gene modelling are often used to mimic PD in animals, and some research groups have used these approaches to explore the neuroprotective potential of carnosine supplementation. These studies found that pre-treatment with carnosine: (i) attenuated apoptosis and upregulated antioxidants malondialdehyde, catalase, and nitrite content in rats injected with 6-hydroxydopamine (6-OHDA), which causes apoptosis in dopaminergic neurons [115], (ii) dose-dependently alleviated the decrease in striatal glutathione peroxidase, retained superoxide dismutase (SOD), decreased proinflammatory cytokines TNF-α, IL-1β, and IL-6, and suppressed inducible nitric oxide synthase (iNOS) mRNA expression in mice treated with 1-methyl-4-phenyl-1,2,3,6-tetrahydropyridine (MPTP), a neurotoxin for dopaminergic neurons [116], and (iii) improved metabolism of dopamine and serotonin in striatum and the frontal lobe in rodents injected with MPTP [117]. In a genetic mouse model of PD characterized by overexpression of α-synuclein, two months of intranasal carnosine intake increased gene transcription of mitochondrial complexes I, IV, and V, protein levels of mitochondrial complex V, and mitochondrial maximal and complex IV-driven respiration [118], which counteracts the well-recognized mitochondrial dysfunction in PD [119]. Further extending this evidence, the carnosine precursor β-alanine was shown to improve motor symptoms of PD and increase extracellular levels of GABA [120] as well as dopamine in substantia nigra [121].

Another protective mechanism of carnosine in neurodegenerative diseases was proposed by Hipkiss [122] by regulating methylglyoxal. Methylglyoxal is a by-product of glycolysis able to induce protein glycation [123] as well as react with dopamine forming neurotoxic metabolites, which can impair mitochondrial function [124]. Animal studies have shown that methylglyoxal levels can be increased following a high glycemic index diet [125] and higher methylglyoxal levels were also found in erythrocytes from patients with T2D [126], which provides an explanation for the link between unhealthy diet and metabolic/neurodegenerative diseases. Conversely, carnosine has been shown to downregulate methylglyoxal and could, therefore, mitigate the neurotoxic effects of this by-product [122].

### 4.3. Psychiatric Disorders

#### Mood Disorders

The most common mood disorder is depression, which is a pathological state of mood accompanied with common feelings of helplessness, hopelessness, worthlessness, and anxiety, which is a feeling of apprehension and fear commonly accompanied by physical symptoms such as palpitations, sweating, and feelings of stress [127]. Rodent models are frequently used to simulate human psychiatric disorders and offer a useful advantage to assess the levels of key neurotransmitters involved in the regulation of mood. Mood disorders share mitochondrial dysfunction, which is germane to the bioenergetics enhancement capacity of carnosine [128,129]. Administration of high dose carnosine was also shown to preserve aging-induced down regulation of the serotonergic system in specific brain regions implicated in the regulation of mental function and mood [130]. The effects of carnosine on mood may depend on dosage as shown in a study of rodents injected with different doses of carnosine, where higher doses produced anxiety-like effects and medium-doses had anxiolytic effects [131]. Acute administration of carnosine as well as chicken breast extract (where carnosine is one of the major components) decreased immobility time in a forced swimming test in rats, which is an index of depression-like behaviour. This effect was accompanied by decreases in hippocampal concentrations of 3-methoxy-4-hydroxyphenylglycol, which is a major metabolite of norepinephrine [132].

Carnosine precursors L-histidine and β-alanine were also investigated in a number of animal studies with regard to mood regulation. Similar to carnosine, peripheral administration of L-histidine dose-dependently induced anxiety-like behaviour in mice [133,134]. On the other hand, two weeks of low dietary intake of L-histidine resulted in lower histamine content in the cortex and midbrain, reduced histamine release from hippocampal neurons and induced anxiety-like behaviour, even though social interaction, memory function, and locomotor activity were not affected [33]. This observation is in agreement with the finding that disruption of the gene coding enzyme, histidine decarboxylase, which catalyses the production of histamine from L-histidine, induces anxiety-like behaviours in mice [135]. Anxiolytic effects of L-histidine, therefore, appear to depend on the histaminergic pathway and activation of H_1_ receptors, since anxiolytic-like behaviours were attenuated with pretreatment with H_1_, but not with H_2_ or H_3_ receptor antagonists [133].

Plasma β-alanine levels were shown to be increased in models of rodents with depression [136] and intracerebroventricular administration of this amino acid that inhibited exploratory behaviour and motility in chicks [137]. In contrast, a diet supplemented with β-alanine induced anxiolytic-like behaviour in mice under acute stressful conditions with concomitant reductions in 5-hydroxyindoleacetic acid (a major metabolite of serotonin) in the hippocampus [138]. Similarly, 30 days of β-alanine supplementation attenuated anxiety behaviour and augmented neurothrophin BDNF expression in both young rats and older rats [139] and increased carnosine content in the rat brain [140].

### 4.4. Summary of Evidence from Animal Studies

Taken together, current evidence from animal studies in neurological disorders suggests that carnosine effectively reduces infarct volume in rodent models of permanent and transient cerebral ischemia in a dose-dependent fashion. These effects are likely mediated by stimulating the expression of growth factors that play a role in neurogenesis, as well as via anti-inflammatory properties and enhanced anti-oxidant and anti-apoptotic capacity. However, additional studies are needed to further elucidate the specific molecular mechanisms behind these effects. In mice models of ASD, higher dietary intake of histidine was shown to ameliorate behavioural impairments through its effects on the mTOR signaling pathway. Furthermore, carnosine has the potential to counteract formation and aggregation of pathological proteins, which is a hallmark of neurodegenerative disorders. In addition, carnosine also improved cognitive disturbances in animals with impairment in glucose metabolism through amelioration of oxidative stress and neuroinflammation, attenuation of astrogliosis, and improvement of cholinergic function. These metabolic benefits of carnosine can, thus, also positively affect the pathophysiology of AD. In animal PD models, pre-treatment with carnosine as well as β-alanine effectively alleviated oxidative stress, inflammatory damage, and dopaminergic neuron loss, and improved mitochondrial function, which reduced motor symptoms and normalized GABA and dopamine levels. In psychiatric disorders, animal studies pointed out carnosine involvement in the regulation of mood behaviour through the histaminergic system as well as via the effects of its precursors, which seem to be concentration-dependent.

## 5. Human Observational Studies of the Role of Carnosine in Brain-Related Disorders

### 5.1. Neurological and Neurodevelopmental Disorders

#### 5.1.1. Autism Spectrum Disorder

Most human cross-sectional and longitudinal studies assessing the levels of carnosine or its peptides in neurological and neurodevelopmental disorders have been conducted in children with ASD, whose carnosine levels as well as levels of its precursors were reduced in urine [141], whereas plasma levels of β-alanine and histidine were found to be higher and carnosine levels were lower when compared to healthy, age-matched controls [142,143]. One hypothesis for these differences is that patients with ASD have higher β-alanine, which results from a gut reaction between ammonia and propionic acid. After crossing the BBB, β-alanine in the brain blocks the receptor sites for GABA, which leads to GABA overproduction as a main inhibitory brain neurotransmitter [144]. In favour of this argument is the fact that the structure of β-alanine is structurally analagous to GABA so β-alanine may serve as a partial GABA antagonist [144]. The potential involvement of carnosine precursors in ASD is further supported with the finding that a model, which included histidine and β-alanine, helped discriminate ASD and intellectual disability in children [145]. This observation was supported by another study that found substantial nutritional and metabolic differences between children with ASD and their healthy-matched controls. Furthermore, amino acid disturbances correlated with disease severity [146].

#### 5.1.2. Multiple Sclerosis

Patients with multiple sclerosis have increased plasma histidine and decreased alanine levels compared with patients with other inflammatory demyelinating diseases [147]. Reductions in serum carnosinase activity have also been proposed as a potential explanation for the impaired exercise capacity, excessive post-exercise fatigue, and reduced muscle contractile function that are usually present in multiple sclerosis [94,148]. It is, therefore, not surprising that individuals with multiple sclerosis had reduced skeletal muscle carnosine content and 10-day exercise training did not restore it [94].

#### 5.1.3. Epilepsy and Hemorrhage

Epilepsy was associated with reduced serum L-histidine levels [149] and its alterations were linked to seizure initiation and propagation in epileptic patients. Histidine has been proposed as a CSF biomarker of subarachnoid hemorrhage complications, with one cross-sectional study finding that CSF levels were significantly increased and correlated with the occurrence of delayed cerebral vasospasms in patients after subarachnoid hemorrhages [150]. Levels of homocarnosine, a putative inhibitory neuromodulator, are higher in the human brain when compared to other animals. The anti-epileptic vigabatrin increased homocarnosine in human CSF in a dose-responsive manner, and seizure control improved with increasing homocarnosine concentrations [151].

### 5.2. Neurodegenerative Disorders

#### 5.2.1. Alzheimer’s Disease

Using a metabolomic approach, increased levels of alanine were identified in AD as well as in amyotrophic lateral sclerosis (ALS), with decreased levels found in PD [152]. Compared with age-matched and sex-matched controls, patients with probable AD (pAD) displayed significant changes in free amino acids and dipeptides involved in neurotransmission, anti-oxidation, and urea metabolism, including reduced plasma carnosine levels [153]. This reduced carnosine may partially explain the decreased anti-oxidant capacity observed in AD, and likely reflects limited dietary intake of carnosine, depletion of carnosine stores due to AD pathology, or decreased endogenous carnosine biosynthesis [153]. Furthermore, carnosinase activity was lower in cases of mixed dementia compared to AD [154] and carnosinase content is recognized as a CSF biomarker for early stages of AD [155]. Similar observations have been made for histidine, where serum levels decreased with the progression of mild cognitive impairment (MCI), which is a stage between the expected cognitive decline of normal aging and the more serious decline of dementia, towards AD [156]. CSF levels were seen as biomarkers for AD progression [157]. Based on these findings, one can conclude that levels of carnosine as well as its rate-limiting precursor, L-histidine, may be altered in AD and could potentially serve as useful biomarkers of disease progression even though more evidence is required.

#### 5.2.2. Parkinson’s Disease

A case-control study [158] of 31 patients with PD found lower levels of carnosine precursors histidine and alanine in the CSF of these patients compared with 45 age-matched and sex-matched controls. The CSF/plasma ratio of many amino acids (including alanine) was also decreased in PD. Therefore, it is possible that PD may contribute to dysfunctional transport of neutral and basic amino acids across the BBB [156]. Another study found 2-fold to 3-fold over-expressed levels of cytosolic non-specific dipeptidase 2 (CNDP2), which is a carnosine degrading enzyme, in substantia nigra of three patients with PD compared with three controls [159]. Compared with healthy controls, patients with idiopathic PD exhibit alterations in several metabolic pathways, including an upregulation of urine histidine and other metabolites in the histidine metabolism pathway [160].

### 5.3. Psychiatric Disorders

#### 5.3.1. Major Depressive Disorder

The diagnosis of many psychiatric disorders lacks reliable methods and is based on symptomatology. A few cross-sectional studies have utilised metabolomic and/or proteomic approaches to explore novel biomarkers and, thus, develop more specific diagnostic tools. These studies found that plasma levels of β-alanine, but not L-histidine or anserine, were lower in patients with MDD treated with selective serotonin reuptake inhibitors (SSRIs) compared with healthy controls [161], but no differences were observed in treatment-resistant patients [162]. Five to six weeks of anti-depressive treatment did not affect any of these amino acids [161,162], even though histidine and glycine concentrations explained 32.2% of variance in the treatment-induced change in MDD measured by the Hamilton Depression Rating Scale [162]. Moreover, a panel of six urinary metabolite biomarkers (including β-alanine) was able to distinguish between commonly misdiagnosed MDD and bipolar disorder with a reliability score of around 90% [163].

#### 5.3.2. Schizophrenia

Schizophrenia is a chronic heterogeneous brain disorder often diagnosed in early adulthood but persisting throughout life, with severe psychopathological, cognitive, and behavioural perturbations [164]. It affects 0.5–1% of the population [165]. Some observational studies suggest that β-alanine and L-histidine may be associated with schizophrenia. Compared with 216 healthy controls, blood histidine levels were significantly lower in 265 patients with stable schizophrenia [166]. Both histidine and β-alanine were also lower in 38 antipsychotic-naïve individuals after their first psychotic episode compared with 36 mentally-healthy controls [167]. In these patients with newly diagnosed schizophrenia, seven months of antipsychotic treatment reversed serum histidine and alanine levels up to levels of control subjects [167]. Plasma histidine levels have also been associated with a risky schizophrenia genotype [166] and alanine with clinical manifestation of schizophrenia [168].

### 5.4. Summary of Evidence from Human Observational Studies

Alterations in amino-acids metabolism and natural antioxidants including carnosine and its compounds seem to have an important role in the pathophysiology of neurological, neurodevelopmental, and neurodegenerative disorders as demonstrated in observational studies. Possible explanations include dysfunction in the circulation-brain transport, increased capacity of its degrading enzymes, or higher excretion rates via urine. The levels of carnosine precursors β-alanine and L-histidine seem to be altered in the circulation of patients with depressive disorders or schizophrenia. In addition, long-term appropriate treatment regimens could likely restore their blood concentrations.

## 6. Human Randomized Controlled Trials of the Role of Carnosine in Brain-Related Disorders

Detailed description of the randomized placebo-controlled trials (RCTs) mentioned in this part is summarized in Table S1.

### 6.1. Neurological and Neurodevelopmental Disorders

#### Autism Spectrum Disorder

Three double-blind RCTs examined the effects of carnosine supplementation in children with ASD. A pioneering study by Chez et al. [169] in 31 children with ASD showed that eight weeks of carnosine supplementation (800 mg daily) decreased autism severity scores compared with placebo, especially behaviour, socialization, and communication sub-scores. Improvements in receptive language and impression of change (as reported by parents) were also observed [169]. This is in contrast with another RCT of the same duration and outcome measure in 43 children, which found no effect on autism scores, perhaps due to the use of a lower dosage of carnosine (500 mg daily). However, improvements in sleep difficulties were reported [170]. A third RCT of 70 drug-naïve children with ASD revealed that 10 weeks of carnosine supplementation (800 mg daily) as an add-on to antipsychotic treatment (risperidone) was effective in reducing hyperactivity and noncompliance (the latter sub-score was reduced by 33.1%), but there was no change in the primary outcome of irritability, as well as in extrapyramidal symptoms [171].

### 6.2. Neurodegenerative Disorders

#### 6.2.1. Cognitive Functions and Alzheimer’s Disease

Several RCTs have explored the effects of supplements containing anti-oxidative and/or anti-inflammatory agents on cognitive performance and the progression of cognitive decline. A few of these have reported promising results using either L-histidine, β-alanine, pure carnosine, a combination of carnosine and anserine, or a combination of carnosine with other agents. The only RCT that examined the effects of pure carnosine [172] was performed in a specific population of individuals diagnosed with Gulf War illness with typical symptoms of cognitive dysfunction, fatigue, and widespread pain. In this case, three months of supplementation (500, 1000, and 1500 mg of carnosine increasing at 4-week intervals) improved executive function and diarrhea associated with irritable bowel syndrome [172]. Conversely, another RCT with only two weeks of supplementation with the carnosine precursor, L-histidine (1.65 g daily), reported improvements in reaction time, clear thinking, and attentiveness in 20 men with a chronic fatigue syndrome [173]. Even short supplementation periods with relatively high doses of β-alanine (6-12 g/day for 12 to 30 days) were shown to improve cognitive performance under stressful conditions in 18 soldiers [174] as well as under simulated sustained military operations in 19 recreationally-active males [175]. Improved exercise performance and elevated muscle carnosine content were also reported in these studies [174,175], but with no change in brain carnosine content [174] or in circulating inflammatory markers [175]. Similar findings were observed in an RCT of 12 elderly volunteers, where supplementation with 2.4 g of β-alanine daily for four weeks eliminated exercise-induced declines in executive functions [176]. However, intervention of the same duration and even higher dosages (6.4 g of β-alanine daily) did not influence cognitive functions before or after acute bouts of exercise in 19 young, well-trained cyclists [177].

More consistent findings have been reported in RCTs using co-supplementation of anserine with carnosine. Cognitive improvements, or at least preservation of certain cognitive domains, were noted in 51 individuals with early stages of cognitive impairment [178], 68 carriers of the highly risky APOE e4 allele genotype for AD [179], and in 31 and 39 cognitively healthy elderly [180,181], following 3–12 months of supplementation with 0.5–1 g of anserine/carnosine (at 2:1 or 3:1 ratio). These changes were accompanied with improvements in brain network connectivity [180] and cerebral blood flow as well as decreased secretion of proinflammatory markers [181]. Ingestion of carnosine-containing formulas also resulted in promising cognitive outcomes in RCTs of cognitively intact elderly [182] and patients with moderate probable AD [183] as well as improved reaction time and attentiveness [184,185] or mood [186] in young, healthy volunteers.

Currently, three other ongoing registered RCTs explore the effects of carnosine supplementation on cognitive outcomes. Two of them involve individuals with prediabetes or type 2 diabetes and who are overweight or obese. Another ongoing RCT combines carnosine supplementation in a dose of 2 g/day with aerobic-strength exercise training in elderly individuals with subjective cognitive decline and/or MCI.

#### 6.2.2. Parkinson’s Disease

Only one pilot RCT [187] has explored the efficacy of carnosine in patients with PD, and found that one month of carnosine supplementation (1.5g daily) increased efficiency of standard PD treatment, improved clinical symptomatology, and enhanced anti-oxidative activity [187]. One more ongoing RCT combines carnosine supplementation with exercise training in patients with PD.

### 6.3. Psychiatric Disorders

#### 6.3.1. Schizophrenia

Two RCTs [188,189] evaluated the effects of carnosine supplementation in patients with schizophrenia prompted by findings from cell and animal studies that show involvement of carnosine in the glutaminergic system and in regulation of N-methyl-D-aspartate (NMAD) and GABA receptors [29,75]. Both trials included similar populations (63 and 75 middle-aged patients with chronic schizophrenia on stable antipsychotic treatments) and dosage regimens (2 g/day carnosine). Additionally, the trial with a shorter duration (eight weeks) reported improvements in total scores as well as negative symptom sub-scores for schizophrenia [188], whereas the trial with a longer duration (three months) found no effect on these scores. However, improvements in several cognitive domains were reported [189]. Both studies found no effects of carnosine on depressive symptoms in patients with schizophrenia [188,189]. An ongoing RCT explores one month of carnosine supplementation in a dose of 2 g/day in combination with cognitive training on cognitive functions in patients with schizophrenia.

#### 6.3.2. Attention-Deficit/Hyperactivity Disorder

The most common neurodevelopmental condition in children, Attention-deficit/hyperactivity disorder (ADHD), is characterised by inattentiveness along with increased impulsivity and hyperactivity, and affects 3% to 7% of children [190]. To date, one RCT has examined carnosine supplementation (800 mg/day for eight weeks) as adjunctive therapy in previously drug-naïve children with ADHD [191]. In this case, carnosine led to modest but significant improvements in clinical symptoms of the disease (especially in the inattention subscale) as assessed by parents, but not by teachers [191].

#### 6.3.3. Obsessive-Compulsive Disorder

Obsessive compulsive disorder (OCD) is among the most disabling conditions worldwide and manifests as unwanted intrusive thoughts and repetitive behaviors [192]. Early research implicated serotonin as the major neurotransmitter involved in OCD pathogenesis. However, glutamate has more recently emerged as an important excitatory neurotransmitter in this pathway [192]. In one RCT [193], authors hypothesized that patients with OCD may benefit from carnosine on the grounds that carnosine was shown to upregulate GLT-1 and, thus, reduce glutamate levels astrocytes [75] as well as in extracellular space [194]. One month of carnosine supplementation as an adjuvant therapy led to reduced total scores as well as obsession and compulsion sub scores on OCD severity ratings [193]. However, larger studies are required to draw more reliable conclusions regarding the effects of carnosine in OCD and its underlying mechanisms.

#### 6.3.4. Behaviour

Two interesting trials have examined the effects of carnosine on various aspects of behaviour, literacy-related skills, and mood. A Japanese trial in 87 healthy office workers [195] showed that a supplement drink containing carnosine (200 mg daily) along with a computerized cognitive behavioural therapy program reduced self-reported tension and anxiety compared to the placebo. Another RCT in Finland investigated the effects of a supplement containing carnosine on literacy-related skills in children [196]. Compared with the placebo, supplements containing omega-3 fatty acids and carnosine (400 mg daily for 90 days) had no effects on any of the outcomes including reading and spelling, language skills, arithmetical skills, and attention [196].

#### 6.3.5. Fatigue

Fatigue is a common symptom in patients with psychiatric disorders [197] and the diagnosis of chronic fatigue syndrome and other brain-related disorders frequently overlap [198]. Considering the beneficial effects of exercise intervention on fatigue [199], a few studies examined the effects of carnosine as an exercise enhancer on fatigue as one of the outcomes in RCTs mentioned above. Two trials conducted in study populations with confirmed diagnosis of chronic fatigue reported contradictory results [172,173]. While, in patients with Gulf War illness, three months of carnosine supplementation had no effect on fatigue [172]. Only two weeks of supplementation with L-histidine (1.65 g daily) improved fatigue scores in men with chronic fatigue syndrome [173]. Similarly, two weeks of supplementation with another carnosine precursor, β-alanine, improved resistance against fatigue during sustained military operation in recreationally active adults [175] and a drink containing carnosine together with a computerized cognitive behavioural therapy program reduced self-reported fatigue in office workers [195].

### 6.4. Summary of Evidence from Human Randomized Control Trials

Although limited, data from three RCTs in children with ASD suggest that carnosine may be effective in improving core as well as secondary symptoms of ASD either alone or in addition to antipsychotic treatment. In spite of the fact that the trials exploring the effects of carnosine supplementation on cognitive functions were of relatively small sample sizes with short supplementation periods, their outcomes suggest that carnosine may protect against conditions associated with fatigue, mood, or cognitive disorders. However, there is a need for larger and more rigorous human intervention studies to corroborate these findings. Undoubtedly, more RCTs are also needed to support the preliminary, albeit promising, results of carnosine augmenting the efficacy of the standard treatment in patients with PD. Given the discrepant results of RCTs in patients with psychiatric disorders, further well-designed trials with adequate dosage regimens and durations examining carnosine are warranted, especially in the early stages of brain-related conditions.

### 6.5. Limitations

Based on the evidence, carnosine represents a promising and safe supportive therapy of specific brain disorders. It is important to point out that no clinical intervention study reported a significantly higher incidence of side effects in carnosine when compared to the placebo group. However, some studies failed to show positive effects of carnosine supplementation on the primary outcomes (ASD, [170], cognitive functions [177,186], schizophrenia [189], literacy-related skills [196]), and the results of some trials lack consistency. For instance, while two trials reported improvements in the disease severity scores in ASD [168,171], another reported no effect [170]. Another important issue is the duration of carnosine supplementation. There are only two intervention clinical trials with a supplementation period longer than three months. Both of these trials used either a combination of carnosine with anserine [179], or carnosine in combination with other bioactive substances [183], so the dose of pure carnosine was substantially lower (e.g., 250 mg vs. 2 g) compared to studies using carnosine only. Thus, the longer intervention trials with pure carnosine supplementation, in doses that were shown to be effective in shorter trials, are needed to establish the safety profile and long-term efficiency of this dipeptide.

## 7. Conclusions

Several lines of evidence, particularly from cell and animal studies, demonstrate potential effects of carnosine on brain-related disorders and the mechanisms underlying these disorders including anti-oxidative, anti-inflammatory, chelating, anti-apoptotic, and anti-glycating properties. These mechanisms were investigated using animal models of various brain pathologies. However, caution is urged when extrapolating these findings to the human context, especially since the levels of carnosinase are much lower in rodents than in humans [10]. Furthermore, dosages of carnosine used in animal studies usually far exceed physiological concentrations in humans. Nevertheless, provisional human studies have shown promising results of supplementation with carnosine or its precursors in specific cognitive functions, improvements in core or secondary symptoms of ASD, schizophrenia, fatigue related disorders, ADHD, and OCD or augmentation of standard treatment of PD. Well designed and adequately powered trials in these disorders are warranted. Despite promising results from studies exploring the effects of carnosine and its derivatives on health, one should bear in mind that the development and pathophysiology of neurodegenerative, neurological, and psychiatric disorders are strongly affected by various risk factors including lifestyle habits, and the supplementation with dietary supplements is not sufficient to compensate for the lack of physical activity and/or the unhealthy diet.

## Supplementary Materials

The following are available online at https://www.mdpi.com/2072-6643/11/6/1196/s1. Table S1: Detailed description of the human randomized controlled trials.
